# Axial length to corneal curvature radius ratio is negatively correlated with choroidal blood flow in myopic children

**DOI:** 10.3389/fopht.2024.1540410

**Published:** 2025-01-06

**Authors:** Yurong Ye, Zizhong Hu, Na Su, Yeyu Shen, Songtao Yuan

**Affiliations:** ^1^ Department of Ophthalmology, BenQ Medical Center Affiliated to Nanjing Medical University, Nanjing, China; ^2^ Department of Ophthalmology, The First Affiliated Hospital of Nanjing Medical University, Nanjing, China

**Keywords:** OCTA, axial length to corneal curvature radius ratio (AL/CR), choroidal blood flow, myopia, children, axial length (AL)

## Abstract

**Background:**

The pathophysiologic mechanisms underlying early-onset myopia remain unclear; in this study, we investigate the pathogenesis by examining the interrelationships between axial length to corneal curvature radius ratio ( AL/CR ) and choroidal blood flow.

**Methods:**

This cross-sectional study included 202 eyes from myopic children, categorized into 141 eyes with mild myopia, 47 eyes with moderate myopia, and 14 eyes with high myopia. Optical coherence tomography angiography (OCTA) was used to measure choroidal blood flow perfusion within a 6 mm × 6 mm area of the macular region, divided into nine subareas based on ETDRS partitioning: macular fovea, nasal side 1, superior 1, temporal side 1, inferior 1, nasal side 2, superior 2, temporal side 2, and inferior 2. Data on corneal curvature and ocular axial length were collected to calculate the AL/CR , with equivalent spherical lens power, gender, and age gathered for group comparisons, and the correlation between AL/CR and choroidal blood flow perfusion volume was analyzed.

**Results:**

AL/CR was significantly negatively correlated with choroidal blood flow perfusion ( P<0.001 ). Linear regression and mediation analyses indicated that for each unit increase in choroidal blood perfusion volume in nasal region 1, AL/CR decreased by an average of 0.421 units. This relationship is mediated by several factors, with axial length serving as a key mediator.

**Conclusion:**

AL/CR correlates with choroidal blood flow perfusion, indicating a link between refractive biological parameters and ocular blood circulation. Myopia is an ischemic eye condition that warrants attention to fundus microcirculation changes in myopic children.

## Introduction

In recent years, the rising incidence of myopia has made it a global public health issue (1, 2), particularly among adolescents, children, and individuals who engage in prolonged close-up visual tasks. Epidemiological surveys indicate a yearly increase in childhood myopia worldwide, especially in East Asia, where the prevalence approaches 50% in some regions (3). Myopia not only affects children’s learning and quality of life but also increases the risk of high myopia and associated complications in adulthood, including retinal detachment, glaucoma, and myopic macular degeneration (4). Previous studies have shown that axial length (AL) is a crucial biological parameter affecting the eye’s refractive state (5, 6). With increasing myopia severity, the eye’s axial length gradually increases. However, axial length alone is insufficient to accurately predict refractive state, as it does not account for corneal curvature(CR). During early myopia development, corneal curvature may increase to compensate for axial elongation. When axial elongation exceeds a certain threshold, corneal flattening can no longer compensate for refractive changes, potentially leading to myopia. Grosvenor first identified a positive correlation between AL and CR, noting that increases in AL are accompanied by proportional increases in CR (7). As AL gradually increases, parallel light converges in front of the retina, with surrounding hyperopic defocus prompting a gradual transition to a myopic refractive state. Therefore, the AL/CR ratio can, to some extent, indicate the refractive status in myopic patients. A recent domestic study involving adolescents and children aged 4–18 years indicated that corneal compensation reaches its upper limit when AL/CR≥3 (8, 9). This ratio is typically below 3 in individuals without myopia, whereas most myopic individuals have an. exceeding 3. Higher ratios correlate with more severe myopia. Thus, the AL/CR ratio can serve as a rapid assessment tool for myopia in adolescents and children, in conjunction with non-dilated pupils optometry and visual acuity testing, as well as a screening method for myopia progression.

Recent studies indicate that changes in choroidal thickness and blood flow are closely related to myopia development (10). As myopic diopter increases and the eye axis elongates, choroidal thickness gradually decreases. Studies suggest that changes in choroidal thickness are not only key indicators of myopia development but may also play a role in regulating eyeball growth and myopia progression. Increased choroidal thickness may help slow myopia progression (11), whereas choroidal thinning may be linked to its rapid progression. Choroidal thinning may result from mechanical stretching due to axial elongation, leading to reduced choroidal blood flow and subsequent retinal ischemia and hypoxia. Additionally, reduced choroidal blood flow may lead to scleral ischemia and hypoxia (12, 13), affecting eyeball growth and myopia development. The application of optical coherence tomography angiography (OCTA) allows for high-resolution, noninvasive evaluation of the retinal and choroidal microvascular structures and blood flow (14). With OCTA, researchers have observed a decrease in the vascular density of the retina and choroid and a decrease in choroidal blood flow in patients with high myopia (15). These changes may relate to myopia development and pathological ocular changes.

The AL/CR ratio is a key indicator for assessing the eye’s refractive state. In myopic children, changes in AL/CR often closely correlate with increasing myopia. Choroidal blood flow and thickness are critical parameters reflecting fundus microcirculation. In myopic children, abnormal choroidal blood flow and thickness changes may be closely linked to myopia onset and progression. The AL/CR ratio, choroidal thickness, and choroidal blood flow are significant topics of research within the field of ophthalmology. However, there is a scarcity of studies examining the correlation between AL/CR and choroidal thickness and blood flow in myopic children. Investigating the distribution characteristics of ocular biological parameters—such as axial length (AL), AL/CR, choroidal thickness, and choroidal blood flow—in myopic children and understanding their relationship with myopia is critical. In this paper, the correlation between AL/CR and choroidal blood flow in children with myopia was evaluated by using a novel statistical analysis method, which provides a new perspective for understanding the pathophysiological mechanism of myopia, aiming to reveal how these parameters affect the occurrence of myopia in children, and whether they can also be an important clinical indicator for evaluating myopia status and myopia prevention and control in children.

## Materials and methods

### Study design and subjects

The research subjects were myopic children who visited the optometry clinic of Nanjing Mingji Hospital affiliated with Nanjing Medical University from December 2022 to June 2024. There were a total of 202 subjects, and data from the right eye was selected for all of them, with a total of 202 eyes included in the study. Each enrolled subject obtained written informed consent to participate.

Inclusion criteria: Participants were aged between 6 and 12, in good health without systemic or ocular diseases, underwent mydriasis with tropicamide, had refraction≤−0.50 DS , corrected visual acuity ≥ 1.0 in both eyes and had normal eye movements.

Exclusion criteria: 1. Systemic underlying diseases; 2. Organic eye lesions (e.g., keratitis, conjunctivitis, trichiasis); 3. Orthokeratology lens wear; 4. Opaque refractive media affecting image acquisition (e.g., congenital cataracts, severe vitreous opacity); 5. Strabismus, amblyopia, or poor fixation ability; 6. Patients undergoing retinal red light therapy.

Data were categorized into three groups based on spherical equivalent (SE) after mydriatic refraction with tropicamide: mild, moderate, and high myopia. The groups were defined as follows: Mild myopia: −3.0D<SE≤−0.50D ; Moderate myopia: −6.0D<SE≤−3.0D ; High myopia: SE≤−6.0D .

### Measurement of ocular biological parameters

Visual acuity, intraocular pressure, comprehensive optometry, and fundus photography were performed by an experienced optometrist. A slit lamp examination was conducted by a senior physician to exclude conditions that might affect the results of this study, such as eyelid trichiasis, conjunctivitis, keratitis, keratoconus, and congenital cataracts.

The ocular optical measurement instrument (Anshikang ASK-Swan600, China) employs partial coherence interferometry (PCI) to measure the axial length of the eye and corneal curvature.

The axial length is typically measured from the center of the cornea to the distance between the optic nerve and the foveal center of the retina, with the unit of measurement being millimeters (mm).

Corneal curvature refers to the degree of curvature of the cornea, describing its shape. ${\text{K}}_{1}$ denotes the corneal curvature value along the line where the cornea is relatively flat, while ${\text{K}}_{2}$ indicates the corneal curvature value in the direction of greater convexity, representing flat K and steep K, respectively. The unit of measurement for corneal curvature is diopter (D).

To avoid the influence of mydriasis on measurement results, the examinee is instructed to undergo a biometry examination prior to the mydriasis examination. Each examinee undergoes five measurements, and the average value is recorded. All examinations are conducted by the same experienced examiner.

AL/CR is the ratio of the axial length of the eye to the average radius of corneal curvature. The calculation method is as follows:


$$\text{Average corneal curvature radius}=\frac{337.5}{\left({\text{K}}_{1}+{\text{K}}_{2}\right)/2}$$

$$\text{AL}/\text{CR}=\frac{\text{axial length}}{\text{average corneal curvature radius }}$$

**Figure 1:**
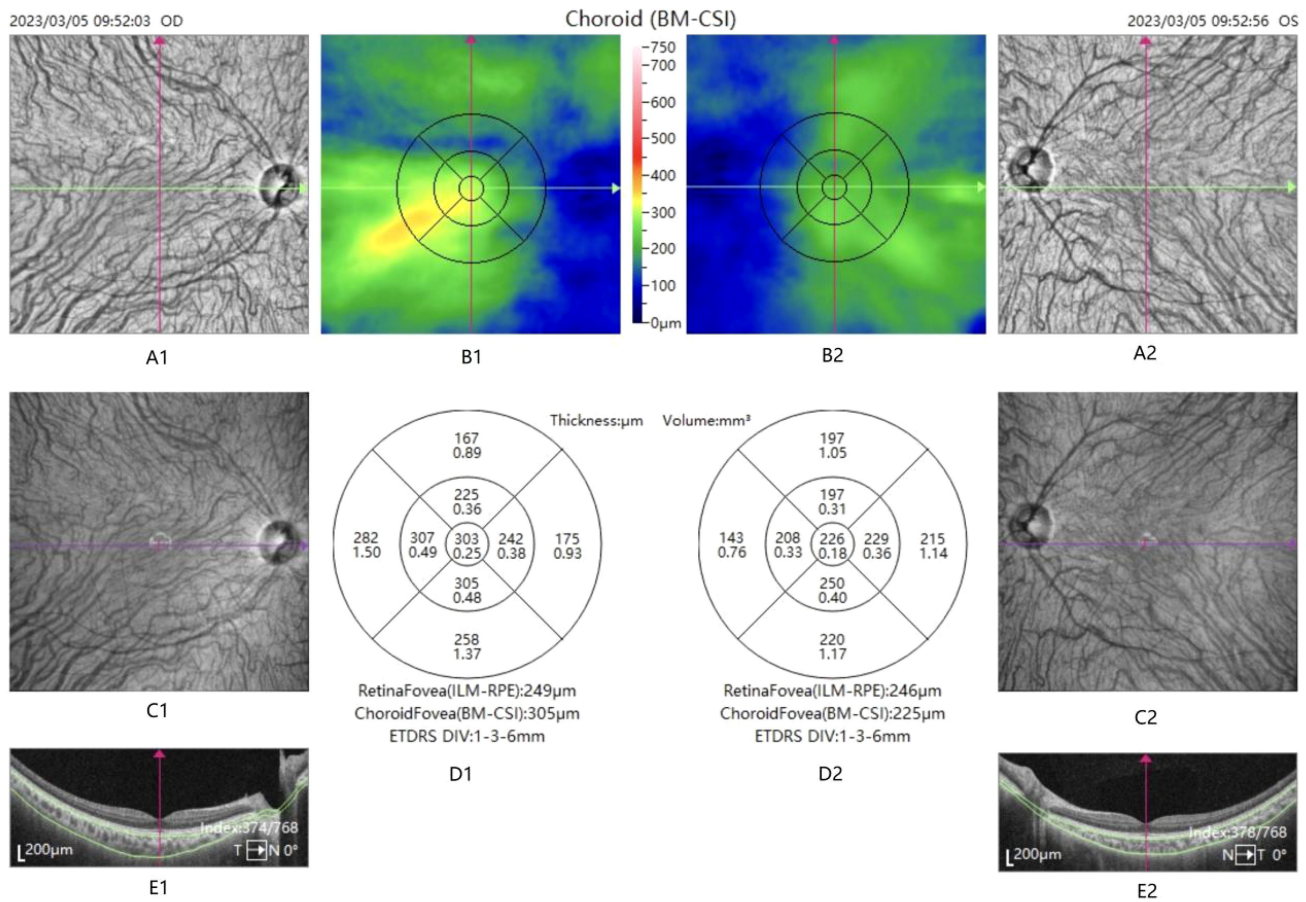
OCTA-acquired choroidal thickness and choroidal blood flow report of the macular region.

OCTA (TowardPi, China) measures choroidal blood flow. Optical Optical Coherence Tomography Angiography (OCTA) is a novel ophthalmic imaging technique that directly observes the blood flow of the retina, choroid, and optic nerve under non-invasive conditions. Utilizing long wavelengths and high-speed scanning, TowardPi OCTA provides an in-depth analysis of the retinal and choroidal microvascular systems, as well as quantitative assessments of blood flow. OCTA was employed to image choroidal blood flow in the macula of all patients. The scan area was divided according to the ETDRS zone, with three concentric circles having diameters of 1 mm, 3 mm, and 6 mm drawn around the fovea, which were defined as the inner ring, the outer ring, and the fovea. Each ring encompasses four areas—upper, lower, temporal, and nasal—and is divided into a total of nine subregions. These nine regions are designated in an anticlockwise direction from the inside out: macular fovea, nasal side 1, superior 1, temporal side 1, inferior 1, nasal side 2, superior 2, temporal side 2, and inferior 2. (See **Figure 1**) The unit of measurement for choroidal blood flow perfusion volume is cubic millimeters (mm³).

A1, A2, C1, C2: enface structure chart. B1,B2: choroidal thickness map. D1,D2: divided into 9 subareas based on ETDRS partitioning: macular fovea, nasal side 1, superior 1, temporal side 1, inferior 1, nasal side 2, superior 2, temporal side 2, and inferior 2. The upper data is the choroidal thickness and the lower data is the choroidal blood flow perfusion volume. E1, E2: OCT Structure chart.

To ensure that mydriasis did not affect the measurement results, the doctor conducted the OCTA examination prior to inducing mydriasis. All examinations were performed by an experienced specialist examiner. The final results were comprehensively evaluated by an experienced specialist physician, and images with blurring or positional shifts during scanning were excluded from consideration.

### Statistical analysis

All the data of each group were statistically analyzed using SPSS 26.0 software, and the measurements were expressed as x ± s . Comparisons between groups were conducted using the ANOVA test, and Pearson correlation analyses were performed on the AL/CR and choroidal blood flow perfusion volume across different divisions. Based on the results of the correlation analysis, linear regression analysis and intermediary effect analysis were performed on the AL/CR and choroidal blood perfusion volume in zone 1 of the nasal side. A p−value<0.05 was considered statistically significant.

## Results

A total of 202 myopic children aged 6 to 12 years were included in this study, with only data from the right eye selected for enrollment, resulting in a total of 202 eyes. Among the participants, 85 were boys (42.6% of the total) and 117 were girls (57.4% of the total). The age distribution was as follows: 30 children aged 6 to 7 years, comprising 12 boys and 18 girls; 80 children aged 8 to 9 years, including 32 boys and 48 girls; and 117 children aged 10 to 12 years, consisting of 41 boys and 76 girls. These results indicate that the prevalence of myopia is higher among girls than boys in all age groups. (See **Table 1**).

**Table 1:** Sex and age as a percentage of the total number.

			age			total
			6 to 7 years old	8 to 9 years old	10 to 12 years old	
sex	boy	count	12	32	41	85
		Percentage of total age	40.0%	40.0%	44.6%	42.1%
	girl	count	18	48	51	117
		Percentage of total age	60.0%	60.0%	55.4%	57.9%
total		count	30	80	92	202
		Percentage of total age	100.0%	100.0%	100.0%	100.0%

Myopic children were categorized into three groups according to spherical equivalent (SE): the mild myopia group consisted of 141 children, accounting for 69.8% of the total, with 53 boys and 88 girls. The moderate myopia group comprised 47 children, representing 23.2% of the total, including 23 boys and 24 girls. The high myopia group included 14 children, accounting for 6.9% of the total, comprising 9 boys and 5 girls. The majority of children with myopia have mild myopia; however, a certain proportion of children exhibit high myopia. In the mild and moderate myopia groups, the incidence is higher among girls, while in the high myopia group, the incidence is higher among boys. (See **Table 2**).

**Table 2:** Gender as a percentage of the total number grouped according to myopia severity.

			myopia severity			total
			mild myopia	high myopia	moderate myopia	
sex	girl	count	88	5	24	117
		Percentage of myopia severity	62.4%	35.7%	51.1%	57.9%
	boy	count	53	9	23	85
		Percentage of myopia severity	37.6%	64.3%	48.9%	42.1%
total		count	141	14	47	202
		Percentage of myopia severity	100.0%	100.0%	100.0%	100.0%

The mean values of axial lengths for the mild myopia group, moderate myopia group, and high myopia group were 24.19 ± 0.34 mm, 25.21 ± 0.67 mm, and 26.45 ± 0.78 mm, respectively, with statistically significant differences between the groups (P < 0.001).

The mean values of AL/CR in the mild myopia, moderate myopia, and high myopia groups were 3.09 ± 0.08, 3.26 ± 0.07, and 3.33 ± 0.06, respectively, with statistically significant differences between the groups ( P<0.001 ). Both axial length and AL/CR exhibited a pronounced upward trend with the increasing severity of myopia.

The mean values of choroidal blood flow perfusion volume in the macular fovea for the mild myopia, moderate myopia, and high myopia groups were 0.208 ± 0.047, 0.187 ± 0.047, and 0.156 ± 0.265, respectively, with statistically significant differences between the groups ( P<0.001 ). As the degree of myopia increases, the choroidal blood flow volume gradually decreases. (See **Tables 3**, **4**).

**Table 3:** Mean values of axial length, AL/CR , and macular fovea choroidal perfusion after grouping by myopia severity.

myopia severity		axial length	AL/CR	macular fovea choroidal perfusion
mild myopia	mean values	24.189	3.094	0.208
	number of cases	141	141	141
	standard deviation	0.739	0.075	0.047
high myopia	mean values	26.446	3.333	0.156
	number of cases	14	14	14
	standard deviation	0.782	0.058	0.027
moderate myopia	mean values	25.207	3.263	0.187
	number of cases	47	47	47
	standard deviation	0.667	0.069	0.047
total	mean values	24.582	3.149	0.200
	number of cases	202	202	202
	standard deviation	0.981	0.114	0.048

**Table 4:** ANOVA results of the mean axial, AL/CR , and macular fovea choroidal perfusion after grouping by myopia severity.

			Sum of squares	Degree of freedom	Mean square	F	Significance
axial * myopia severity	between groups	groups	88.802	2	44.401	84.309	0.000
	Intra-group		104.803	199	0.527		
	total		193.605	201			
AL/CR * myopia severity	between groups	groups	1.531	2	0.765	143.555	0.000
	Intra-group		1.061	199	0.005		
	total		2.591	201			
macular fovea choroidal perfusion * myopia severity	between groups	groups	0.044	2	0.022	10.421	0.000
	Intra-group		0.424	199	0.002		
	total		0.468	201			

“*” represent significance levels of 10% respectively.

The correlation test results between AL/CR and macular fovea choroidal perfusion (r=-0.315, P<0.001), nasal 1 choroidal perfusion (r=-0.323, P<0.001), temporal 1 choroidal perfusion (r=-0.314, P<0.001), superior 1 choroidal perfusion (r=-0.264, P<0.001), inferior 1 choroidal perfusion (r=-0.162, P<0.001), nasal 2 choroidal perfusion (r=-0.302, P<0.001), temporal 2 choroidal perfusion (r=-0.303, P<0.001), superior 2 choroidal perfusion (r=-0.195, P< 0.001), and inferior 2 choroidal perfusion (r=-0.178, P<0.001).

According to the results of the Pearson correlation analysis, we can find a negative correlation between the AL/CR and the choroidal perfusion volume, and the largest correlation coefficient and the strongest correlation with the choroidal perfusion volume of the nasal side 1 region.

**Figure 2:**
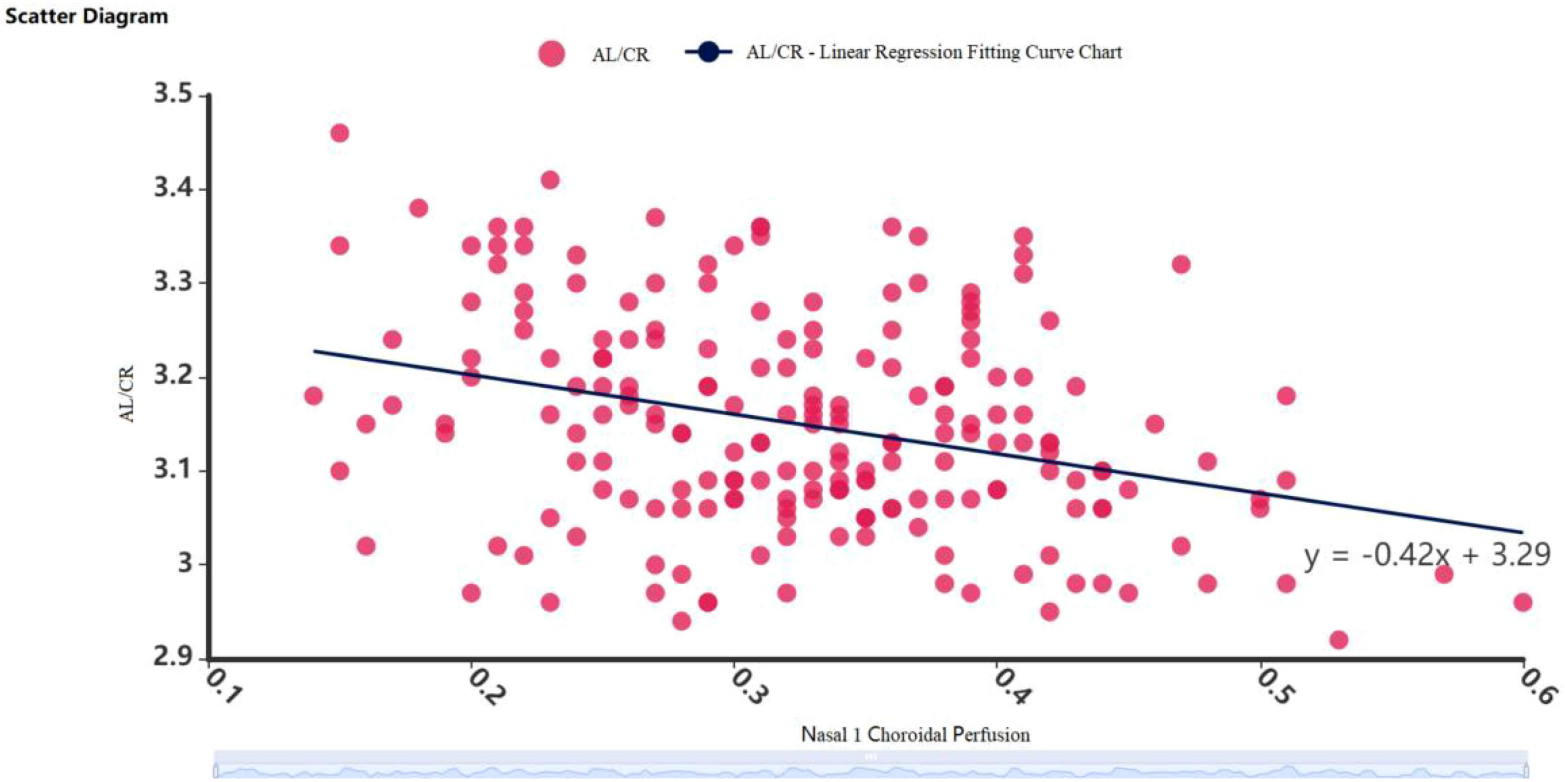
Scatter diagram of linear regression between AL/CR and choroidal blood flow perfusion volume in the nasal side 1 region.

Based on the results of Pearson correlation analysis, a linear regression model was established by choosing choroidal blood perfusion in region 1 of the nasal side as the predictor variable and AL/CR as the dependent variable. The adjusted R-squared value was 0.100, F = 23.274, P < 0.001 , and the model as a whole was statistically significant. The regression coefficient value of AL/CR and choroidal blood perfusion volume in region 1 of the nasal side was -0.421 ( t = −4.824, P < 0.001 ), indicating that when choroidal blood perfusion volume increases by 1 unit, the AL/CR will decrease by an average of 0.421 units. (See **Figure 2**).

To further investigate the relationship between choroidal blood perfusion volume, AL/CR , axial length, corneal curvature, and equivalent spherical lens power, we designated AL/CR as the dependent variable (Y), nasal 1 choroidal blood perfusion volume as the independent variable (X), and equivalent spherical lens power, axial length, corneal curvature ${\text{K}}_{1}$ , and corneal curvature ${\text{K}}_{2}$ as mediating variables to construct a mediation model. (See **Table 5**).

**Table 5:** Summary results of the intermediate effect test process.

effect	item	Effect value	Standard error	t	P	Lower bound of 95% confidence interval	Upper limit of 95% confidence interval
Direct effect	nasal 1 choroid blood perfusion volume=>AL/CR	-0.002	0.003	-0.694	0.488	-0.009	0.004
Indirect effect	nasal 1 choroid blood perfusion volume=>axial length	-5.43	0.699	-7.764	0.000***	-6.809	-4.051
	nasal 1 choroid blood perfusion volume=>equivalent spherical	0.869	1.236	0.703	0.483	-1.568	3.307
	axial length=>equivalent spherical	-1.409	0.11	-12.856	0.000***	-1.625	-1.192
	nasal 1 choroid blood perfusion volume=>corneal curvature ${\text{K}}_{1}$	0.558	0.724	0.771	0.442	-0.869	1.985
	axial length=>corneal curvature ${\text{K}}_{1}$	-1.382	0.087	-15.945	0.000***	-1.553	-1.211
	equivalent spherical=>corneal curvature ${\text{K}}_{1}$	-0.53	0.041	-12.786	0.000***	-0.612	-0.448
	nasal 1 choroid blood perfusion volume=>corneal curvature ${\text{K}}_{2}$	1.17	0.56	2.089	0.038**	0.066	2.274
	axial length=>corneal curvature ${\text{K}}_{2}$	-0.274	0.101	-2.703	0.007***	-0.473	-0.074
	equivalent spherical=>corneal curvature ${\text{K}}_{2}$	-0.185	0.043	-4.282	0.000***	-0.271	-0.1
	corneal curvature ${\text{K}}_{1}$ =>corneal curvature ${\text{K}}_{2}$	0.804	0.055	14.639	0.000***	0.696	0.912
	axial length=> AL/CR	0.129	0.001	206.43	0.000***	0.128	0.13
	equivalent spherical=> AL/CR	0.001	0	3.184	0.002***	0	0.001
	corneal curvature ${\text{K}}_{2}$ => AL/CR	0.035	0	73.141	0.000***	0.034	0.036
	corneal curvature ${\text{K}}_{2}$ => AL/CR	0.038	0	88.132	0.000***	0.037	0.039
Total effect	nasal 1 choroid blood perfusion volume=> AL/CR	-0.421	0.087	-4.824	0.000***	-0.594	-0.249

*** and ** represent significance levels of 1% and 5% respectively.

In the chain mediation analysis, this study systematically explored the pathway of the nasal side 1 blood flow volume on the AL/CR, the sequence of mediating variables (axial length, refractive power, corneal curvature K1, and corneal curvature K2). First, the direct effect analysis showed that the nasal side 1 blood flow volume had no significant direct effect on the axial ratio (effect size=-0.002, P=0.488), indicating that there was no strong direct correlation between the two. This finding emphasizes the importance of mediating variables in explaining the relationship between the two.

Then, an indirect effect analysis revealed the mediating pathway. Nasal 1 blood flow volume had a significant indirect negative impact on the AL/CR through the mediating effect of the eye axis (effect size = -5.43, P < 0.001), indicating that the eye axis is the mediator through which nasal 1 blood flow volume affects the axis ratio. At the same time, the negative impact of nasal 1 blood flow volume on the eye axis was also significant (effect size = -5.43, P < 0.001), further supporting the validity of this pathway.

However, the indirect effect of nasal side 1 blood flow volume on the AL/CR through refractive index (equivalent spherical power) was not significant (effect size = 0.869, P = 0.483), although refractive index was significantly influenced by eye axis (effect size = -1.409, P < 0.001). This suggests that although there is a strong correlation between eye axis and refractive index, refractive index does not play a significant mediating role in the pathway from nasal side 1 blood flow volume to AL/CR.

Additionally, the corneal curvature K1 and K2 also played an important role in the chain mediation effect. The direct effect of nasal side 1 blood flow volume on corneal curvature K1 was not significant (effect size=0.55, P=0.442), but it had a significant positive effect on corneal curvature K2 (effect size=1.17, P=0.038). There was a significant strong positive correlation between corneal curvature K1 and K2 (effect size=0.804, P<0.001), and both were significantly positively correlated with the AL/CR (effect size=0.035 and 0.038, P<001). These findings suggest that corneal curvature is not only an important component of the axial structure of the eye, but also an important mediator of the indirect effect of nasal side 1 blood flow volume on the AL/CR.

Finally, the total effect analysis showed that the total effect of nasal side 1 blood flow volume on the ratio of axial length to curvature was a significant negative effect (effect size=-0.421, P<0.001). This result was the result of the combined action of multiple mediating variables, with the axial length and corneal curvature playing a crucial mediating role.

## Discussion

The present study reveals the complex relationship among these parameters through detailed measurements and analyses of sex, age, AL/CR , and choroidal blood flow perfusion in myopic children aged 6 to 12 years. Although the myopic population predominantly exhibits mild myopia, a notable percentage of high myopia is now observed. Furthermore, the prevalence of myopia is relatively higher in girls for moderate and mild myopia, while it is significantly greater in boys for high myopia. The study indicates that AL/CR is more pronounced in the group of myopic children, showing a corresponding increase with the progression of myopia. This finding suggests that AL/CR is not merely a static assessment criterion; rather, it represents an evolving process that accurately describes the trajectory of myopia. The changes in choroidal blood perfusion volume also provide valuable reference information. We found that as AL/CR gradually rises, the choroidal perfusion volume exhibits a decreasing trend. The early stage of myopia is characterized not only by changes in the axial length of the eye but also by alterations in the overall configuration of the eye and the role of depth regulation (16). This phenomenon can be explained biologically; the elongation of the eye’s axial length may cause the choroid to become thinner (17, 18) and more susceptible to stretching, thereby reducing the vascular mobility space and compromising blood transport pathways. Additionally, the contraction of the ciliary muscle during near work may transmit tension to the posterior pole of the eye, contributing to the elongation of the eye axis (19). In this process, the ciliary muscle may become hypertrophic (20), competing for a portion of choroidal blood perfusion, which exacerbates choroidal ischemia and hypoxia, leading to scleral hypoxic remodeling (12)and further elongation of the eye axis.

Our study found the strongest negative correlation between AL/CR and choroidal blood perfusion volume in nasal zone 1, corroborating the findings reported by Luo et al. In their investigation, Luo et al. observed that choroidal blood vessel distribution diminished progressively with increasing degrees of myopia, with the most pronounced alterations occurring in the nasal region of the macula, as well as the temporal and inferior areas surrounding the optic disc (21). Our results provide further validation of their conclusions. The mechanisms underlying these observations may be related to the compromised integrity of the papillon-macular bundle. Jonas et al. demonstrated that as axial length increases, both the parapapillary gamma zone (the peripapillary sclera devoid of overlying choroid, Bruch’s membrane, and deep retinal layers) and the delta zone (characterized by the absence of blood vessels with diameters exceeding 0.5 mm within the gamma zone) expand, accompanied by a gradual reduction in choroidal perfusion (22). Consequently, the papillon-macular tract may play a pivotal role in the onset and progression of myopia.

This study also further elucidated the significant specificity of the AL/CR ratio in myopic children within a clinical setting. With the progression of myopia, the AL/CR ratio exhibited a marked upward trend. This phenomenon suggests that the AL/CR ratio can serve not only as a critical reference index for the diagnosis of myopia but also as a dynamic indicator of myopia progression, independent of traditional optometric methods to some extent. Thus, this research provides a novel scientific foundation for the early diagnosis and prevention of myopia. Furthermore, the study highlighted the significant correlation between choroidal blood flow and the degree of myopia. As myopia worsens, there is a noticeable decreasing in choroidal blood flow. This relationship underscores the interaction between refractive biological parameters of the eye and ocular hemodynamics. The findings are further supported by the observation that an increase in the AL/CR ratio coincided with reduction in choroidal blood flow, reinforcing the interconnectedness of these factors in the context of myopia progression.

The study focused on children aged 6 to 12 years, a critical period for the development of myopia. During this time, mild myopia is more prevalent, whereas moderate and high myopia may require a longer duration to develop or may be influenced by specific genetic factors. In the sample, the proportion of children with moderate and high myopia was small, which means the findings may primarily reflect the characteristics of those with mild myopia. As a result, the study may not fully represent the entire population of myopic children, potentially impacting the generalizability and external validity of the results.

The data collection and analysis in this study were primarily based on a single-center cross-sectional study design. Although a correlation was identified between AL/CR and choroidal blood flow, the exact causality of this relationship remains uncertain. Future studies should consider expanding the sample size and enhancing collaborative efforts among multiple research centers, adopting a longitudinal research strategy to track changes in ocular parameters over various time periods. This approach will help ensure that the research results are more broadly applicable and trustworthy. Furthermore, due to the technical limitations of the examination equipment, it was not possible to determine whether some of the blood with reduced choroidal perfusion was redistributed into the ciliary muscle. It is hoped that more advanced measurement tools and optimized experimental procedures will become available in the future to observe and analyze the pathophysiological processes associated with reduced choroidal perfusion.

The present study focused on the relationship between AL/CR and choroidal blood flow in myopic children aged 6 to 12 years. As myopia progressed, AL/CR exhibited a significant upward trend, while choroidal blood perfusion gradually decreased. This trend suggests a close relationship between the refractive biological parameters of the eye and fundus blood flow, indicating that decreased choroidal perfusion in the fundus is evident even in the early stages of myopia, which can be classified as an ischemic fundus disease. By integrating multiple factors such as the refractive biological parameters of the eye and the condition of fundus blood flow, we can gain a more comprehensive understanding of the biological mechanisms underlying childhood myopia and provide robust scientific support for the development of more effective myopia prevention and control strategies.

This study highlights the significant influence of biological parameters, such as AL/CR and choroidal blood flow, on the development of myopia. It involves a detailed analysis of these parameters in myopic children aged 6 to 12. In clinical practice, AL/CR can serve as a valuable screening indicator when combined with measurements of eye axis length and corneal curvature, enabling healthcare professionals to more accurately evaluate the risk of myopia in children. Additionally, by regularly monitoring the structural and functional status of the choroid, clinicians may be able to detect and address potential microcirculation issues in myopic patients in a timely manner.

## Data Availability

The raw data supporting the conclusions of this article will be made available by the authors, without undue reservation.
